# Supplementary material for: Comprehensive Analysis of Metabolites and Biological Endpoints Providing New Insights into the Tolerance of Wheat Under Sulfamethoxazole Stress
Source: Int J Mol Sci. 2025 Apr 30;26(9):4257. doi: 10.3390/ijms26094257 (PMC12072142; doi:10.3390/ijms26094257)
Supplement: Supplementary file 1 [file ijms-26-04257-s001.zip › Supplementary Material.pdf]

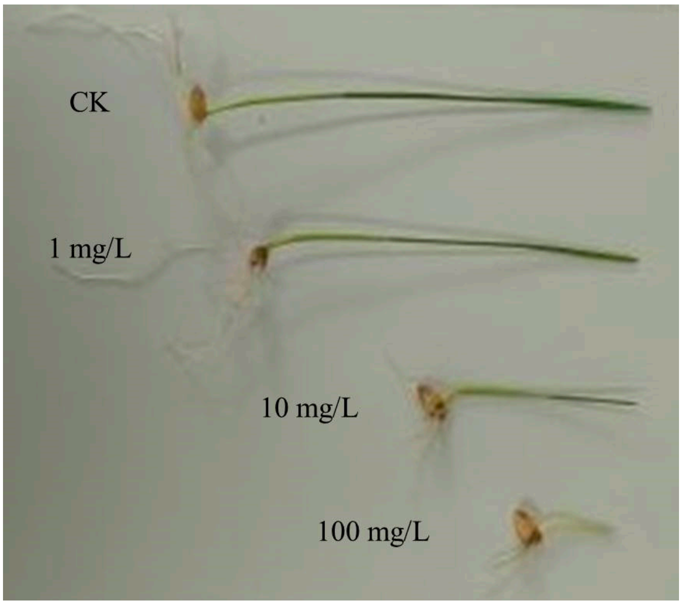

Figure S1 Seedling phenotypes.

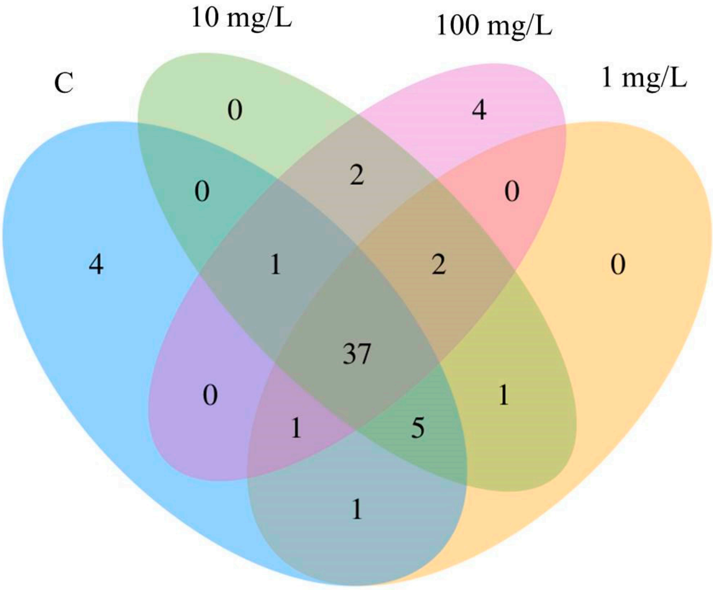

Figure S2 Venn analysis of metabolites.

Table S1 The relationships between main metabolites and root fresh weight

Metabolites	VIP	Coefficients	Correlation
4-Aminobutanoic acid	1.4152	-0.04092	-0.953
Allose	1.44458	0.037386	0.999
Arabinopyranose	1.41872	-0.03799	-0.996
Fructose	1.44146	-0.04091	-0.936
Glucose	1.44776	-0.03638	-0.946
Homoserine	1.36978	-0.0406	-0.934
Lactose	1.43935	0.041044	0.951
Serine	1.43915	-0.03775	-1.000
Sucrose	1.41063	-0.03416	-0.975

Table S2 The relationships between main metabolites and ROS level

Metabolites	VIP	Coefficients	Correlation
4-Aminobutanoic acid	1.35159	0.036027	0.930
Allose	1.46301	-0.03746	-0.995
Arabinopyranose	1.45887	0.038088	0.994
Fructose	1.30889	0.034139	0.902
Glucose	1.35872	0.033544	0.926
Homoserine	1.32447	0.03586	0.912
Lactose	1.34032	-0.03526	-0.923
Serine	1.46375	0.037751	0.997
Sucrose	1.44339	0.035969	0.977
Xylitol	1.35131	0.033493	0.904

Table S3 The relationships between main metabolites and root length

Metabolites	VIP	Coefficients	Correlation
2-Amino-2-deoxyhexose	1.37841	0.036364	0.938
3-Hydroxybutyric acid	1.37841	0.036364	0.938
Adenine	1.40232	-0.03556	-0.950
Alanine	1.33964	0.034628	0.907
Asparagine	1.42207	-0.03711	-0.967
Butanoic acid	1.37841	0.036364	0.938
Glucose	1.43377	-0.0358	-0.975
Glyceric acid	1.38844	-0.03642	-0.948
Glycolic acid	1.3296	-0.03541	-0.905
Leucine	1.36447	0.036097	0.928
Myo-Inositol	1.3667	0.034013	0.925
Threonic acid	1.37841	0.036364	0.938
Threonine	1.39882	-0.03554	-0.955

Table S4 The relationships between main metabolites and SOD activity

Metabolites	VIP	Coefficients	Correlation
Allose	1.39712	-0.03447	-0.960
Arabinopyranose	1.33121	0.032424	0.915
Glucose	1.44667	0.036105	0.993
Myo-Inositol	1.3152	-0.03164	-0.901
Phosphoric acid	1.31413	-0.03435	-0.904
Serine	1.37595	0.033782	0.946
Sucrose	1.43297	0.0361	0.985
Threonine	1.41695	0.036642	0.973

Table S5 The relationships between main metabolites and POD activity

Metabolites	VIP	Coefficients	Correlation
4-Aminobutanoic acid	1.48755	0.045413	0.988
Adenine	1.35118	0.038039	0.906
Allose	1.4651	-0.0382	-0.971
Arabinopyranose	1.46459	0.040196	0.970
Fructose	1.48745	0.044263	0.991
Glucose	1.42652	0.035386	0.950
Homoserine	1.45981	0.046054	0.969
Lactose	1.49591	-0.04481	-0.995
Myo-Inositol	1.4436	-0.04195	-0.964
Serine	1.46925	0.039106	0.974
Sucrose	1.3954	0.03273	0.925
Xylose	1.44061	-0.04628	-0.956

Table S6 The relationships between main metabolites and CAT activity

Metabolites	VIP	Coefficients	Correlation
5-Oxoproline	1.31027	0.03472	0.901
Allose	1.44372	-0.03612	-0.984
Arabinopyranose	1.40919	0.035195	0.958
Glucose	1.39881	0.034604	0.957
Phosphoric acid	1.37311	-0.03589	-0.944
Serine	1.43326	0.035824	0.976
Sucrose	1.46079	0.036956	0.999
Threonine	1.35463	0.034339	0.933
Xylitol	1.35779	0.035674	0.931
